# Sex differences in the initiation of antithrombotic therapy in patients with atrial fibrillation in Germany

**DOI:** 10.1007/s00392-025-02757-7

**Published:** 2025-10-07

**Authors:** Jamschid Sedighi, Mark Luedde, Boris Dinov, Philipp Bengel, Priyanka Boettger, Christian Tanislav, Samuel Sossalla, Karel Kostev

**Affiliations:** 1 Medical Clinic I, Cardiology and Angiology, Justus-Liebig-University, Klinikstraße 33, 35392 Giessen, Germany; 2 Christian-Albrechts-University of Kiel, Kiel, Germany; 3 Department of Geriatrics and Neurology, Diakonie Hospital Jung Stilling, Siegen, Germany; 4 Department of Cardiology, Kerckhoff-Clinic, Bad Nauheim, Germany; 5 German Center for Cardiovascular Research (DZHK), Partner Site Rhine-Main, Rhine-Main, Germany; 6 Epidemiology, IQVIA, Frankfurt, Germany

**Keywords:** Atrial fibrillation, Antithrombotic therapy, Sex disparities, Stroke prevention, Cardioembolic

## Abstract

**Background and aims:**

Despite guidelines recommending antithrombotic therapy for atrial fibrillation (AF) patients, differences in treatment initiation between sex remain an area of ongoing research. This study assesses the association between sex and the likelihood of receiving antithrombotic therapy following an AF diagnosis.

**Methods:**

A retrospective multicenter cohort study was conducted using data from the IQVIA™ Disease Analyzer database, comprising 118 cardiology practices in Germany. Patients ≥ 18 years with a first-time AF diagnosis between 2010 and 2023 were included. The primary outcome was the prescription of vitamin K antagonists, direct thrombin inhibitors, or direct factor Xa inhibitors within 30 days post-diagnosis. Multivariable logistic regression models examined the association between sex and therapy initiation, adjusting for age, CHA₂DS₂-VA Score, and health insurance coverage.

**Results:**

Among 166,005 patients (74,755 female, 91,250 male), female patients were significantly older than their male counterparts, with a mean age of 76.2 years compared to 73.2 years (*p* < 0.001). They however had a similar median CHA_2_DS_2_-VA Score (2). Overall, the proportion of patients with antithrombotic prescriptions were lower in females (13.2%) compared to males (15.5%, *p* < 0.001). Multivariable regression revealed a negative association between female sex and therapy initiation (OR: 0.89; 95% CI: 0.86–0.91, *p* < 0.001).

**Conclusion:**

Our findings indicate notable disparities in the initiation of antithrombotic therapy in relation to sex, thus underscoring the necessity for additional research to comprehend the underlying factors. Consequently, efforts should be concentrated on enhancing adherence to treatment guidelines, addressing sex-specific barriers to therapy, and ensuring equitable access to optimized stroke prevention strategies for patients with AF.

**Graphical Abstract:**

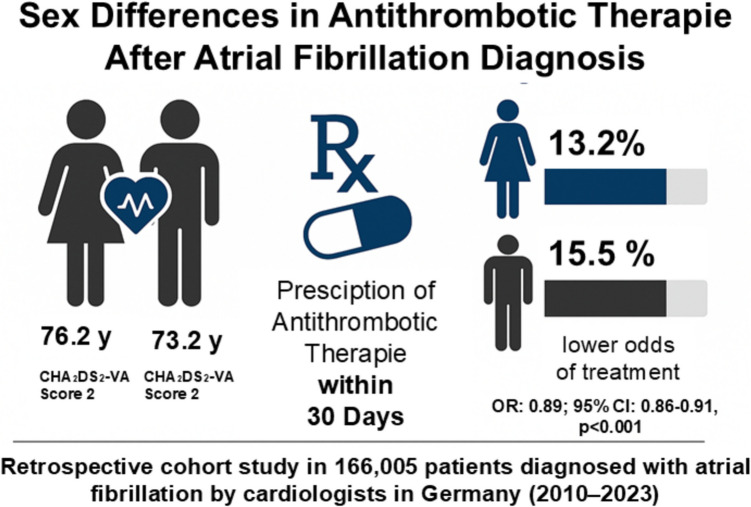

## Introduction

Atrial fibrillation (AF) is the most common sustained cardiac arrhythmia and represents the principal etiological factor in the development of cardioembolic stroke, heart failure, and overall mortality [1]. Given the significant morbidity associated with AF, stroke prevention through antithrombotic therapy remains a cornerstone of management. Current guidelines strongly recommend anticoagulation therapy for patients at an increased risk of thromboembolic events, as stratified by the CHA₂DS₂-VA scoring system [2, 3]. However, despite clear guideline-based recommendations, discrepancies in the initiation of anticoagulation therapy persist in clinical practice.

Sex differences in cardiovascular disease management have been extensively reported, with multiple studies indicating that female patients with AF often receive less aggressive treatment compared to their male counterparts [4]. Women with AF generally have a higher CHA₂DS₂-VASc Score than men due to the inclusion of female sex as a risk factor, resulting in a greater estimated stroke risk [5]. Despite this, research suggests that women are less likely to receive antithrombotic therapy, which may be attributed to physician prescribing behavior, patient-related factors, or broader systemic issues [4, 5].

This study was designed to investigate potential differences in antithrombotic therapy initiation among newly diagnosed AF patients in Germany. By analyzing real-world prescription patterns, we aim to delineate the determinants influencing anticoagulation decisions and identify opportunities for improving adherence to guideline-based therapy. Addressing sex disparities in AF management is crucial for optimizing patient outcomes and ensuring equitable stroke prevention strategies [6].

## Methods

### Database

This retrospective study utilized the Disease Analyzer database (IQVIA™), which includes anonymized data from 3000 general and specialist practices in Germany [7]. The database is representative of outpatient practices and has been employed for several published studies on AF [8, 9]. This analysis included data from all 118 office-based cardiology practices contained in the IQVIA Disease Analyzer database. While the database comprises approximately 3000 general and specialist practices across Germany, it contains only these 118 cardiology practices nationwide, representing the complete population of office-based cardiologists within the dataset. Most participating sites are single-specialty outpatient clinics. General practitioners were deliberately excluded to focus exclusively on therapy initiation by cardiology specialists.

### Study population and outcome

**Fig. 1:**
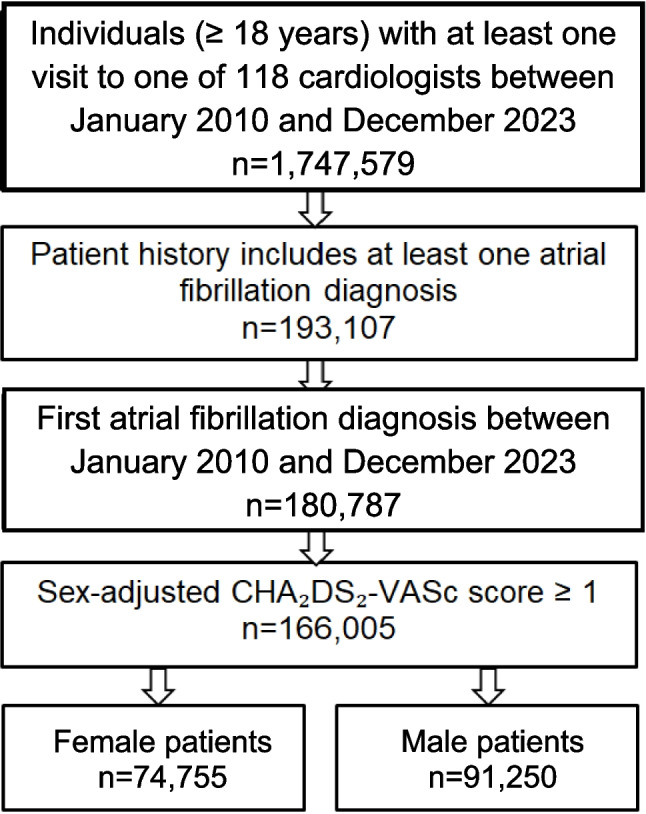
Selection of study patients

The selection of the cohort for this study is depicted in Fig. 1. Patients aged ≥ 18 years with a first-time AF diagnosis (ICD-10: I48.0, I48.1, I48.2, I48.9) documented between 2010 and 2023 and with a sex-adjusted CHA₂DS₂-VASc score (CHA₂DS₂-VA score) ≥ 1 were included. Those with prior antithrombotic therapy or history of stroke were excluded to isolate new treatment decisions. The outcome of interest was the prescription of vitamin K antagonists, direct thrombin inhibitors, or direct factor Xa inhibitors within 30 days of AF diagnosis, which was analyzed by sex. The proportion of treated patients was determined by dividing the number of individuals with active prescriptions by the total number of patients diagnosed with atrial fibrillation.

The Disease Analyzer database does not capture details on AF subtype (paroxysmal, persistent, or permanent), diagnostic method (e.g., ECG, Holter monitoring), or whether the AF was symptomatic or asymptomatic. As a result, analyses stratified by AF type, AF burden, or symptom presentation could not be performed.

Since patients who do not receive therapy from a cardiologist after diagnosis are typically considered lost to follow-up, we opted not to employ Kaplan–Meier methodology, as its use would significantly overestimate the proportion of treated patients.

### Statistical analyses

Baseline characteristics of female and male study patients were compared using Chi-squared tests using Chi-squared tests for categorical variables, the student’s *t*-test for data for continuous data with normal distribution and the Wilcoxon signed-rank test for continuous data that did not follow a normal distribution. Furthermore, Chi-squared tests were used to compare prescription rates between sex across age and CHA₂DS₂-VA groups. Multivariable logistic regression models estimated odds ratios (ORs) and 95% confidence intervals (CIs) for the association between sex and therapy initiation, adjusting for age, CHA₂DS₂-VA Score, and health insurance coverage. A *p*-value of < 0.01 was considered statistically significant.

### Ethics

Our research protocol was evaluated by the local ethics committee of Christian-Albrechts-University (CAU) Kiel, Germany; since we used only anonymized data that could not be traced back to individual persons, it was confirmed that it was not necessary to obtain informed consent from individual patients to participate in the study (file reference D413/21 of the CAU ethics committee).

## Results

The study included a total of 166,005 patients diagnosed with AF, of whom 74,755 were female and 91,250 were male. Female patients were significantly older than their male counterparts, with a mean age of 76.2 years compared to 73.2 years (*p* < 0.001). They however had a similar median CHA_2_DS_2_-VA Score (2). Additionally, females were less likely to have private health insurance (5.2% vs. 10.7%, *p* < 0.001), which may have influenced treatment accessibility (Table 1).

**Table 1:** Baseline characteristics of the study sample

Variable	Proportion among female patients (%) *N* = 74,755	Proportion among male patients (%) *N* = 91,250	*P* value*
Age (mean, SD)	76.2 (8.5)	73.2 (9.7)	< 0.001
Age < 65	6.285 (8.4)	15,631 (17.1)	< 0.001
Age 65–74	21,724 (29.1)	29,780 (32.6)	
Age ≥ 75	46,746 (62.5)	45,839 (50.2)	
Year 2010–2013	13,949 (18.7)	16,549 (18.2)	0.011
Year 2014–2017	21,176 (28.3)	25,679 (28.1)	
Year 2018–2020	19.401 (25.9)	24,123 (26.4)	
Year 2021–2023	20,229 (27.1)	24,899 (27.3)	
Private health insurance coverage	3898 (5.2)	9.787 (10.7)	< 0.001
CHA₂DS₂-VA Score (median, IQR)	3 (2)	3 (2)	0.004
CHA₂DS₂-VA Score: 1–2	30.573 (40.9)	38,476 (42.2)	< 0.001
CHA₂DS₂-VA Score: 3	20.731 (27.7)	22,792 (25.0)	
CHA₂DS₂-VA Score: 4	13,462 (18.0)	16,709 (18.3)	
CHA₂DS₂-VA Score: 5 +	9989 (13.4)	13,273 (14.5)	
Diagnoses documented within 12 months prior to or on index date			
Obesity	10.353 (13.9)	12,427 (13.6)	0.174
Type 2 diabetes	11,685 (15.6)	17,437 (19.1)	< 0.001
Hypertension	45,402 (60.7)	54,118 (59.3)	< 0.001
Dyslipidemia	15,684 (21.0)	22,889 (25.1)	< 0.001
Heart failure	17.801 (23.8)	24,976 (27.4)	< 0.001
Ischemic heart diseases	16,137 (21.6)	32,772 (35.9)	< 0.001
History of stroke	6751 (9.0)	8.539 (9.4)	0.022
History of pulmonary embolism	1137 (1.5)	1180 (1.3)	< 0.001
Venous embolism and thrombosis	1307 (1.8)	1437 (1.6)	0.006
History of gastrointestinal bleeding	104 (0.1)	184 (0.2)	0.002
Drugs prescribed within 12 months prior to or on index date			
Prescription of aspirin	1513 (2.0)	3111 (3.4)	< 0.001
Prescription of clopidogrel	751 (1.0)	1952 (2.1)	< 0.001

Proportions of patients given in % unless otherwise indicated. *SD*, standard deviation; *IQR*, interquartile range

*Chi-squared test for categorical data, Student’s *t*-test for continuous data with normal distribution, Wilcoxon signed-rank test for continuous data without normal distribution

### Antithrombotic prescription rates

Overall, antithrombotic therapy was initiated in 13.2% of female patients and 15.5% of male patients within 30 days of AF diagnosis (*p* < 0.001). Of them, only 11.7% received Vitamin K antagonists since 88.3% received DOAC.

The difference in prescription rates remained significant across all age groups and CHA₂DS₂-VA Score categories.

Prescription rates for each age group include all patients in that category, regardless of whether they met the guideline-based CHA_2_DS_2_-VASc threshold for anticoagulation. Given the high prevalence of comorbidities and age-related risk factors, it is highly likely that the vast majority of patients met the guideline indication, although this analysis was based on age grouping alone.

- Among patients under 65 years, 11.4% of women and 14.0% of men received antithrombotic therapy.
- In the 65–74 age group, the prescription rates were 14.3% in females and 16.2% in males.
- For patients aged 75 and older, therapy initiation was recorded in 13.0% of women and 15.9% of men.

**Fig. 2:**
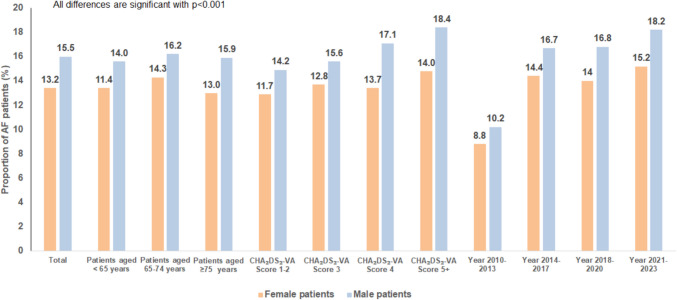
Proportion of AF patients initiated on antithrombotic therapy within 30 days of diagnosis, stratified by sex

When analyzed by CHA_2_DS_2_-VA Score groups:

- In patients with a score of 1–2, the prescription rates were 11.7% for females and 14.2% for males.
- Among those with a CHA_2_DS_2_-VA Score of 3, 12.8% of women and 15.6% of men received treatment.
- Among those with a CHA_2_DS_2_-VA Score of 4, 13.7% of women and 17.1% of men received treatment.
- In the highest risk category (CHA_2_DS_2_-VA Score ≥ 5), therapy was prescribed to 14.0% of females and 18.4% of males (Fig. 2).

Finally, the proportion of antithrombotic therapy increased from period 2010–2013 years (8.8% in women, 10.2% in men) to 2021–2023 (15.2% in women, 18.2% in men).

### Association between sex and therapy initiation

Multivariable logistic regression analysis confirmed a statistically significant association between female sex and lower odds of receiving antithrombotic therapy (OR: 0.89; 95% CI: 0.86–0.91, *p* < 0.001). The disparity was particularly pronounced among patients with a CHA₂DS₂-VA Score ≥ 5 (OR: 0.81; 95% CI: 0.77–0.86), suggesting that even among those at highest stroke risk, females were less likely to receive antithrombotic therapy (Table 2).

**Table 2:** Association between female sex and antithrombotic drug prescriptions in patients with atrial fibrillation monitored in cardiology practices in Germany (multivariable logistic regression models)

Patient group	Odds ratio (95% CI)	*P* value
Total*	0.89 (0.86–0.91)	< 0.001
Patients aged 65 years**	0.87 (0.80–0.95)	< 0.001
Patients aged 65–74 years**	0.96 (0.91–1.00)	0.071
Patients aged ≥ 75 years**	0.85 (0.82–0.89)	< 0.001
Patients with CHA₂DS₂-VA Score: 1–2***	0.93 (0.89–0.97)	0.002
Patients with CHA₂DS₂-VA Score: 3***	0.94 (0.89–0.99)	0.018
Patients with CHA₂DS₂-VA Score: 4***	0.80 (0.75–0.86)	< 0.001
Patients with CHA₂DS₂-VA Score: 5 + ***	0.81 (0.77–0.86)	< 0.001
Year 2010–2013*	0.89 (0.82–0.97)	0.004
Year 2014–2017*	0.89 (0.85–0.94)	< 0.001
Year 2018–2020*	0.87 (0.82–0.92)	< 0.001
Year 2021–2023*	0.85 (0.81–0.90)	< 0.001

*Multivariable logistic regression adjusted for age, CHA₂DS₂-VA Score, and private health insurance coverage

**Multivariable logistic regression adjusted for CHA₂DS₂-VA Score and private health insurance coverage

***Multivariable logistic regression adjusted for age and private health insurance coverage

## Discussion

**Fig. 3:**
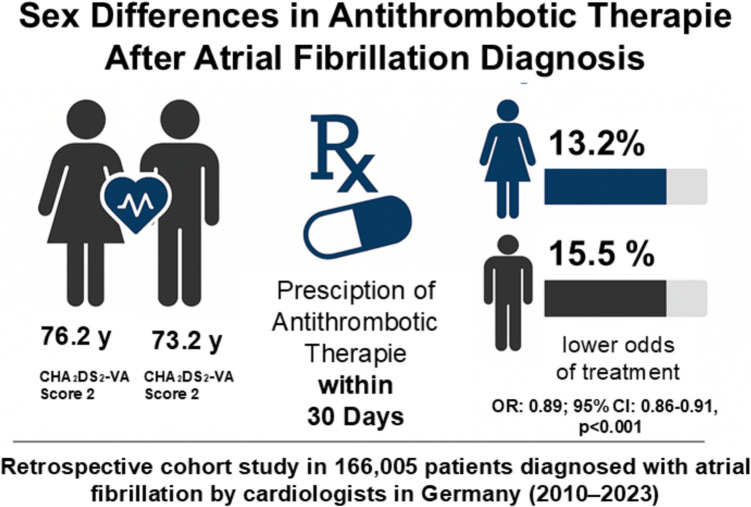
Sex differences in antithrombotic therapy initiation after atrial fibrillation diagnosis in Germany (2010–2023). Data from 166,005 patients diagnosed by cardiologists in 118 outpatient practices. Women were older (mean 76.2 y) than men (mean 73.2 y) but had the same median CHA₂DS₂-VA score (2). Within 30 days of diagnosis, antithrombotic therapy was prescribed in 13.2% of women and 15.5% of men. Multivariable analysis showed lower odds of treatment initiation in women (OR: 0.89; 95% CI: 0.86–0.91, *p* < 0.001)

This study highlights significant sex differences in the initiation of antithrombotic therapy among patients with incident AF. Although clinical guidelines recommend early anticoagulation to reduce thromboembolic risk, women were less likely than men to receive this treatment within 30 days of diagnosis. This disparity was evident across all age groups and CHA_2_DS_2_-VA score categories, with increasing discrepancy for patients considered to be at higher risk (Fig. 3).

### Low rate of antithrombotic therapy initiation

One notable finding of this study is that only 13.2% of female patients and 15.5% of male patients received antithrombotic therapy within the first 30 days following AF diagnosis. Several factors could contribute to this relatively low prescription rate. Besides gaps in knowledge and varying interpretations of treatment protocols, physicians may be cautious about prescribing anticoagulants due to concerns about bleeding risks, particularly in elderly patients or those with multiple comorbidities. Notably, patients with a higher CHA_2_DS_2_-VA score often present an elevated HAS-BLED score, which can lead to hesitancy in prescribing anticoagulants, further complicating the decision-making process as clinicians weigh the benefits of stroke prevention against potential hemorrhagic complications [10, 11].

Another factor may be the budgeting constraints in clinical practice, where financial considerations limit the prescription of anticoagulants, particularly for patients with intermediate CHA₂DS₂-VA scores.

Compared to other European countries with similar healthcare systems, our observed initiation rates of antithrombotic therapy in AF patients are markedly lower. Registry data from Denmark [12] show that OAC adherence in eligible AF patients increased from 53% in 2013 to 78% in 2022. In the Netherlands, registry data indicate persistence rates above 70%, with apixaban users showing up to 84% adherence after one year [13]. In Finland, OAC initiation was recorded in 71.6% of all incident AF patients, with higher rates among high-stroke-risk patients [14]. These cross-country differences are likely to reflect variations in the implementation of guidelines, prescribing habits, reimbursement structures and the division of care responsibilities between general practitioners and specialists. It is essential to understand these systemic and cultural factors in order to address the relatively low initiation rates in Germany.

The role of the treating physician may also contribute to the observed prescription patterns. Our study focuses exclusively on data from cardiology practices, but previous studies suggest that general practitioners play an essential role in initiating antithrombotic therapy for AF patients [15].

Another important consideration is patient decision-making and preferences. Some patients may decline anticoagulation due to concerns about side effects or the need for regular blood monitoring, particularly in the case of vitamin K antagonists [16]. Ensuring patient education and addressing concerns about anticoagulation side effects may help improve adherence to guideline recommendations.

### Differences in prescription rates for CHA₂DS₂-VA scores of three or four

The finding that the prescription rate is low among patients with a CHA_2_DS_2_-VA score of three or four is a particularly striking one. Although current guidelines advocate antithrombotic therapy for these patients, there is considerable variation in prescription practices, which may be due to a lack of clarity amongst medical practitioners regarding the net clinical benefit of treatment. This hesitation may be more pronounced among older patients or those with additional health concerns [17].

### Systemic factors and sex disparities in AF treatment

In addition to individual treatment decisions, systemic factors also influence anticoagulation rates. Regional variations in healthcare implementation, reimbursement policies, and access to specialist care play a role in determining prescription patterns. Research has shown that privately insured patients are more likely to receive guideline-adherent antithrombotic therapy compared to those covered by statutory insurance [18].

The 2024 ESC guidelines introduced a revised version of the CHA₂DS₂-VASc score, now termed CHA₂DS₂-VA, which excludes female sex as a formal scoring component [3]. This revision reflects the recognition that female sex alone does not confer additional stroke risk in the absence of other risk factors, and should instead be regarded as a risk modifier. While previous versions may have led to inconsistent prescribing behavior—particularly in low-risk women—the updated score aims to simplify the decision-making process by clearly identifying truly low-risk patients (i.e., CHA₂DS₂-VA = 0), for whom oral anticoagulation is not recommended.

The continued increase in prescriptions for antithrombotic drugs until 2023 is likely due to a combination of the availability of DOACs, evolving clinical guidelines, improved reimbursement frameworks, increased awareness among the public and professionals, and extensive real-world data confirming their superiority over VKAs in terms of safety, efficacy and patient compliance.

### Understanding sex differences in antithrombotic therapy

Studies have consistently reported that female AF patients are less likely to receive anticoagulation therapy than male patients, despite having a higher overall stroke risk [11, 19, 20]. Several factors may contribute to this discrepancy. One possible explanation is the variation in risk assessment, where certain clinical characteristics in women may influence anticoagulation decisions. Additionally, women are often older at the time of AF diagnosis and have a higher prevalence of comorbidities, potentially influencing prescribing behaviors [17].

The existence of disparities in cardiovascular care access has been demonstrated to be a contributing factor to observed differences in treatment approaches. A higher proportion of female patients are managed in primary care settings as opposed to by specialists, with the treatment approaches of the latter being known to differ [21]. Physician biases, differences in symptom presentation, and variations in patient adherence could further impact anticoagulation decisions [16].

Recent evidence has provided strong justification for re-evaluating the role of female sex in the CHA₂DS₂-VASc score. Yoshimura et al. [22], using a large UK registry, showed that the modified CHA₂DS₂-VA score (excluding the sex component) maintained equivalent predictive performance for thromboembolic events when compared to the original CHA_2_DS₂-VASc score. Importantly, the study suggested that the statistical contribution of female sex to risk discrimination was minimal and likely not clinically meaningful.

Adding to this, Teppo et al. [23] demonstrated that the association between female sex and ischemic stroke has progressively weakened over time and was no longer evident in recent years. Teppo et al. [24] further identified that sex disparities in oral anticoagulant use have persisted over more than a decade, even as treatment options (e.g., DOACs) and clinical guidelines evolved.

Lip et al. [25] have argued that removing the sex component from the CHA₂DS₂-VASc score could help standardize decision-making and reduce ambiguity, particularly in borderline cases.

Taken together, this redefinition may support more consistent risk assessment and prescribing decisions. However, it remains uncertain whether removing female sex from the scoring algorithm will reduce undertreatment in women, especially since sex-based disparities were already present under the previous CHA₂DS₂-VASc system. Therefore, while the CHA₂DS₂-VA score may streamline classification, additional measures, such as clinician education and awareness, are likely necessary to ensure equitable application of guideline-based therapy in clinical practice.

### Limitations

While this study provides valuable insights into sex differences in antithrombotic therapy initiation among AF patients, several limitations should be acknowledged. First, our dataset lacks information on important lifestyle factors such as smoking status, alcohol consumption, and physical mobility. Additionally, due to unavailability of mortality data, we were unable to assess the potential impact of variations in treatment initiation on long-term survival outcomes. In addition, the lack of data on AF subtype, AF burden, and symptom status is a limitation, as these factors may affect a physician’s decision to initiate antithrombotic therapy and could contribute to the low overall prescription rates observed.

Furthermore, it is possible that the prescribing behavior of treating physicians may also influence the observed prescription patterns. Patients in this study are observed exclusively within a single practice; consequently, any prescriptions issued by external physicians are not documented. Consequently, if anticoagulants were prescribed by general practitioners outside of cardiological specialist settings, our data may not fully reflect the complete scope of antithrombotic therapy initiation.

## Conclusion

The findings of this study highlight critical differences in the initiation of antithrombotic therapy among patients with AF in Germany. While clinical guidelines recommend the prescription of anticoagulation in eligible patients, real-world data suggest that prescribing patterns may be influenced by multiple factors beyond clinical risk scores alone. It is essential to understand these variations in order to improve adherence to evidence-based recommendations and optimize patient outcomes. Addressing disparities in antithrombotic therapy requires a multifaceted approach, including clinician education, patient engagement, and healthcare system interventions.

Further research is needed to explore the underlying drivers of these differences and to develop targeted strategies that ensure all patients receive optimal and equitable care. Our findings provide a foundation for future investigations and underscore the importance of continued efforts to bridge the gap between guidelines and clinical practice.

## Data Availability

The data that support the findings of this study are derived from the Disease Analyzer database (IQVIA), which includes anonymized health records from general and specialist practices in Germany. The datasets used and analyzed during the current study are available from the corresponding author on reasonable request.
